# Haloperidol Induced Sudden Cardiac Arrest—Report of a Very Rare Case and Review of Literature

**DOI:** 10.1155/2020/1836716

**Published:** 2020-07-22

**Authors:** Parvez Mohi-Ud-Din Dar, Supreet Kaur, Jogendra Boddeda, Sajid Mohammad Wani

**Affiliations:** ^1^Division of Trauma Surgery and Critical Care, Jai Prakash Narayan Apex Trauma Centre, All India Institute of Medical Sciences (AIIMS), New Delhi, India 110029; ^2^Department of Psychiatry, Sher-I-Kashmir Institute of Medical sciences (SKIMS), Srinagar, Jammu and Kashmir, India 190011

## Abstract

Haloperidol is a typical antipsychotic drug. This drug is still widely used in emergency medicine, psychiatry, and general medicine departments. It is mostly used for acute confusional state, psychotic disorders, agitation, delirium, and aggressive behaviour. Overdose of haloperidol can cause sudden deaths. Cardiopulmonary arrest related to use of haloperidol had been reported in literature as case reports but are very few. No such cases have been reported in India till now. We report a case of cardiac arrest due to the use of haloperidol.

## 1. Introduction

Haloperidol is a typical antipsychotic drug derived from butyrophenone. This drug is still widely used in emergency medicine, psychiatry, and general medicine departments. It is mostly used for acute confusional state, psychotic disorders, agitation, delirium, and aggressive behaviour [1]. In addition to the D2 blockage, haloperidol also inhibits alpha 1 adrenergic receptors. It has weaker effects on muscarinic, cholinergic, and histaminergic receptors [2]. Overdose of haloperidol can cause sudden deaths and had been seen with normal intravenous or oral therapeutic dose [3, 4].

Although clinically significant effects are not common, the cardiovascular effects of antipsychotic drugs include arrhythmia, prolonged QT syndrome, receptor blockage, conduction disorders, sinus node abnormalities, myocarditis, cardiomyopathy, and postural hypotension. Delayed ventricular repolarisation is also one of the most important effects of antipsychotic drugs. Its projection on the electrocardiogram as prolonged QT interval may increase the risk of tachyarrhythmia and sudden cardiac death [5, 6]. Cardiopulmonary arrest related to use of haloperidol has been reported in literature in the form case reports but are very few. No such cases have been reported in India till date. We report a case of pulseless ventricular tachycardia and cardiac arrest related to the use of haloperidol.

## 2. Case

A 62-year-old male was brought by his relatives to emergency department (ED) with alleged history of road traffic injury (RTI). The patient was initially taken to local hospital, where initial treatment was given and referred to our trauma centre. The patient reached our ED 16 hours post injury. On arrival to ED, he had following vital signs.

### 2.1. Primary Survey

Primary survey was as follows: airway—patent, cervical spine stabilized; breathing—spontaneous, respiratory rate 18/min, Spo2-99%, air entry bilateral present, chest compression test was negative; circulation—pulse 112/min, blood pressure 106/74 mmHg, pelvic compression test was negative; disability—GCS was E4V5M6; pupil bilateral normal size, reacting to light, moving all four limbs equally; Focused Assessment with Sonography for Trauma (FAST) tested positive in splenorenal pouch; chest X-ray and pelvis X-ray were normal; and right leg X-ray was showing fracture both bones of the right leg.

On further examination, the left lower limb was cold to touch, pallor, painful, and paraesthesia was present. Pulse was absent in the left anterior tibial artery, posterior tibial artery, popliteal artery, and femoral artery. Routine blood investigations (complete blood count, liver function test, kidney function test, serum electrolytes, and coagulation profile) were normal. Contrast Enhanced Tomography (CECT) of the torso showed grade III splenic injury. CT angiography of lower limbs showed thrombus in the left common iliac artery. The patient was taken to the operation theatre, and left hip disarticulation and external fixation of both bones of the right leg were done. In the postoperative period, the patient was taken to intensive care unit (ICU) for further resuscitation and management. The patient underwent multiple surgeries and was kept in ICU for 34 days. During the course of ICU stay, the patient developed features of psychosis after 32 days in ICU. Psychiatry consultation was sought and was diagnosed delirium. The patient was administered 2.5 milligrams of intravenous haloperidol. Two minutes after giving haloperidol intravenous, the patient developed pulseless ventricular tachycardia with absent carotid and femoral pulses followed by cardiac arrest. Immediate cardiopulmonary resuscitation (CPR) was started as per the Advanced Cardiac Life Support (ACLS) guidelines. Cardioversion was given with 200 joules 4 times, and 1 millilitre adrenaline 1/1000 was also given. The patient was reviewed after 5 minutes of CPR.

## 3. Discussion

There are only a few case reports of life threatening situations and cardiopulmonary arrest in literature due to the use of haloperidol [7, 8]. The potential of causing arrhythmia and sudden death is very low with the haloperidol as compared to drugs like thioridazine. Mehtonen et al. [9] have studied 49 sudden deaths associated with the use of antipsychotic or antidepressant drugs. 28 cases were those who were using thioridazine, while only 6 cases were of those using haloperidol; however, in none of these 6 cases, haloperidol was used alone. 15 out of the 28 thioridazine cases used only thioridazine. It is likely that haloperidol by blocking the potassium ion channels in heart muscle cells (in ventricular myocytes) leads to the decrease of repolarization there by lengthening of action potential duration and QT interval, and arrhythmias [10]. In the present case after giving haloperidol, the patient developed pulseless ventricular tachycardia and tachyarrhythmia. The side effect has occurred after serious trauma, and it most probably exacerbated the situation in our case. In patients on haloperidol treatment, if there are flat T waves in the ECG of the patients U waves and the QTc interval is over 500 milliseconds or the QTc interval is lengthened by 15-20%, the medication should be stopped. In these patients, serum electrolytes like sodium, potassium, and calcium should be checked regularly, and any medicine causing lengthening of QT interval should be avoided.

The American Psychiatric Association's (APA) Diagnostic and Statistical Manual of Mental Disorders- (DSM) IV [11] defined the delirium as a disturbance of consciousness and cognition that develops over a short period of time (hours to days) and fluctuates over time. The different terms which also have been used to describe this syndrome of cognitive impairment in critically ill patients include ICU psychosis, ICU syndrome, acute confusional state, encephalopathy, and acute brain failure [12, 13]. The critical care literature has recently conformed to the recommendations of the APA and other experts that the term “delirium” be used uniformly to describe this syndrome of brain dysfunction.

Haloperidol is still used very often in the treatment of delirium and agitation. The risk of pulseless ventricular tachycardia, prolongation of QT, and cardiac arrest should be taken into account while using this medicine. Serial ECG monitoring of these patients should be done and special precautions should be taken in patients having prolonged QT in ECG. Further research is needed to be done in larger population groups to explain the exact mechanism of pulseless ventricular tachycardia and cardiac arrest.

## Data Availability

Data is available upon reasonable request: Data can be obtained from the corresponding author upon request.
